# Effect of Encapsulation Processes by Freeze and Spray Drying on the Antioxidant Properties of Red Wine from cv. Listan Prieto and Syrah

**DOI:** 10.3390/foods11233880

**Published:** 2022-12-01

**Authors:** Irina Díaz-Gálvez, Gastón Gutiérrez-Gamboa, Andrea Plaza, Anibal A. Concha-Meyer

**Affiliations:** 1 Instituto de Investigaciones Agropecuarias INIA, Centro de Investigación Raihuén, San Javier 3660000, Chile; 2 Escuela de Agronomía, Facultad de Ciencias, Ingeniería y Tecnología, Universidad Mayor, Temuco 4780000, Chile; 3 Centro de Estudios en Alimentos Procesados (CEAP) Conicyt-Regional R19A10001 Gore Maule, Talca 3460000, Chile; 4 Instituto de Ciencia y Tecnología de los Alimentos, Facultad de Ciencias Agrarias y Alimentarias, Universidad Austral de Chile, Valdivia 5090000, Chile

**Keywords:** wine powder, patrimonial variety, antioxidants, phenolic compounds, freeze-drying, spray-drying

## Abstract

Background: Wine antioxidants are linked to cardiovascular disease prevention, thus are highly valued by the healthy food market. The dehydration process removes alcohol and water from wine and allows it to extend its shelf life, while encapsulation can help preserve physical-chemical and antioxidant properties. Moreover, information on the effect of wine drying and encapsulation on non-anthocyanin phenolic compounds is limited in the literature. Methods: Listan Prieto and Syrah (*Vitis vinifera* L.) wines were dehydrated and converted into powder by freezing and spray drying. Powdered wines were subjected to water activity, pH, soluble solids, color, and phenolic compounds analysis. Results: Freeze-drying process produced powdered wines with higher pH than the spray-drying process. Powdered wines made by these processes presented similar water activity and soluble solids. Powdered wines did not show statistical differences in *trans*-resveratrol, hydrocinnamic acids, phloretin, kaempferol, and quercetin content according to their dehydration process. In addition, powdered wines significantly concentrated hydrocinnamic acid and quercetin when compared to non-dealcoholized and dealcoholized wine samples. Conclusions: The results suggest that the dehydration process does not negatively modify the characteristics of the wine, and it retains a significant concentration of phenolic compounds. Therefore, powdered wines have an interesting potential to be used as a natural source of antioxidants for food supplementation.

## 1. Introduction

Moderate red wine consumption has shown scientific evidence that is oppositely associated with cardiovascular disease linked to a significant concentration of phenolic compounds in these kinds of products [1,2,3]. Red wine is recognized as a significant source of phenolic compounds such as phenolic acids, major catechins, resveratrol, and anthocyanins, which are present in grape seeds, skins, and vine stems [4]. Phenolic compound concentration in red wine is influenced by grape variety and vintage [5], in addition to pedoclimatic conditions and vinification practices [6,7].

Listan Prieto (cv. País) is the founding grapevine variety of Latin American viticulture, which was introduced during the Spanish colonization [8,9]. Chilean wine industry used this variety for massive wine production, but since the 80s, several Listan Prieto vineyards were uprooted and some European varieties, such as Syrah, Cabernet Sauvignon and Sauvignon Blanc were extensively introduced by the big companies due to their high enological potential [10,11]. Today, Listan Prieto is still relegated within the national wine industry and the vines are used as rootstock for other commercial varieties when retrofitting a vineyard [12]. In recent years, innovative wines have been developed with Listan Prieto [9], as well as other minority or patrimonial varieties of Chile to increase product market value [13]. The wine industry has made efforts to promote the consumption of Listan Prieto wine by diversifying production [13,14] and interesting sparkling wines have been produced, but the demand for these products is still limited.

The consumption of red wines has been associated with blood pressure reduction in hypertensive persons due to the significant presence of antioxidant compounds [15]. Nevertheless, the ingestion of alcohol must be moderate (1–2 glasses per day) to prevent alcohol-related diseases, which limits its potential as a good source of antioxidants [16]. Therefore, the transformation of wines into raw materials for the food industry emerges as an alternative to add value and manufacture different healthy food and beverage products. 

Protection from light and heat as well as assuring stability of polyphenols over long-term storage conditions can be achieved by encapsulation techniques [16,17]. There are numerous encapsulating agents available for use in food including hydrocolloids such as maltodextrin that offer protection against phenol oxidation, in addition to providing emulsifying properties, low hygroscopicity, and a low economic cost. Currently, the food industry protects the bioactive compounds form oxidation by performing powder encapsulation methods that involve spray drying and freeze-drying technologies, which make products more stable during storage and easy to handle [18,19]. Sánchez et al. [20] observed that maltodextrin encapsulation of a Cabernet Sauvignon wine by freeze drying produced an increase of about 3.7 times the content of phenolic compounds when compared to liquid wine. Also, Álvarez Gaona et al. [21] studied the maltodextrin DE10 encapsulation of Ancellotta red wine by spray-drying and obtained a low-water activity wine powder that contained 33 antioxidant compounds. Furthermore, total anthocyanins and malvidin-3-*O*-glucoside concentration in Ancelotta red wine powder showed no significant variation after 90 days of storage at 38 °C. Currently, there is limited information in the literature on the effect of Listan Prieto red wine encapsulation to protect non-anthocyanin phenolic compounds.

The objective of this work was to study the effect of maltodextrin encapsulation using spray drying and freeze drying on physicochemical, color, total phenolics and non-anthocyanins phenolic compounds in Listan Prieto and Syrah wine to obtain a powder with potential to be used as ingredient in food and/or pharmaceutical applications. 

## 2. Materials and Methods

### 2.1. Raw Materials

Wine samples were provided by the Cooperativa Vitivinícola de Loncomilla (San Javier, Chile) and consisted of twelve-month aging wines from Syrah and Listan Prieto (*Vitis vinifera* L.) varieties elaborated in the 2015 vintage. From each variety were obtained two “burgundy type” 225 L oak barrels. Syrah and Listan Prieto wines reached 14.9 ± 0.42 and 15.3 ± 0.10 of alcohol degree; 3.6 ± 0.5 and 3.5 ± 0.0 of pH; and 3.6 ± 0.6 and 3.0 ± 0.8 of total acidity (g L^−1^ of tartaric acid), respectively. 

### 2.2. Sample Preparation

The wines were dealcoholized and concentrated at 60 °C and 0.2 bar using a pilot scale industrial vacuum condenser (Bigman, Linares, Chile) provided by Elaboradora y Extractora Ecocrea Ltd.a. (Colbún, Chile). Alcohol concentration in the condensed fraction was determined using a Gay-Lussac Alcoholmeter (Boeco, Staufen, Germany) and the concentration process ceased when 40% alcohol volume was reached in the condensed hydroalcoholic phase. Then, samples were encapsulated using maltodextrin DE_15_ (Prinal, Santiago, Chile), which was dissolved in wine to 25% concentration (total weight basis), according to the methodology exposed by Sánchez et al. [20]. 

Freeze drying process was performed using maltodextrin encapsulated dealcoholized wine samples that were distributed into 100 mm diameter Petri dishes with a depth 1 cm and previously frozen at −86 °C for 48 h. Subsequently, the samples were placed in a freeze dryer (Operon, FDU 7024, Gimpo, Republic of Korea) for 24 h with a cold trap temperature of −70 °C and vacuum pressure conditions below 200 μm Hg. The freeze-dried samples were completely pulverized using a ceramic mortar and powdered wine was obtained. 

The spray drying process was performed in a pilot scale spray dryer DR15 (Spray Process, Sao Paulo, Brazil) provided by Elaboradora y Extractora Ecocrea Ltd.a. (Colbún, Chile). Briefly, the process was carried out using 150 L of Syrah and Listan Prieto wine previously dealcoholized and encapsulated samples that were atomized and dried with an inlet temperature of 160 °C and outlet temperature of 70 °C. 

Freeze-dried and spray-dried samples were stored in sealed opaque plastic bags at room temperature. Freeze-drying and spray-drying sample treatments were performed in duplicate.

### 2.3. Physicochemical, Color and Total Phenolic Content Analysis

Soluble solids (°Brix), pH, and water activity (a_w_) were determined in treated wine samples. Soluble solids and pH were evaluated by dissolving 1 g of sample in 9 mL of distilled water using a HI96801 digital refractometer (Hanna Inc., Woonsocket, RI, USA) and a TitroLine easy meter (Schott Inc., Mainz, Germany), respectively. The determination of water activity was performed on powdered samples using a Hygrolab C1 digital hygrometer (Rotronic Instrument Corp., New York, NY, USA) previously calibrated. A color analyzer system (Model RGB-1002, Lutron Co., Ltd., Taipei, Taiwan) with a measurement range of 350 to 750 nm was used to measure the color of powdered samples and color software was used for data interpretation n into CIE Lab color parameters (Adobe PhotoshopElements 11, Adobe Systems, San Jose, CA, USA). Total phenolic compounds were determined on dried and non-dried wine samples by Folin-Ciocalteu method according to Singleton et al. [22] by dissolving 1 g of powdered sample in 9 mL of water in the case of dried samples and results were expressed as gallic acid equivalents (GAE) mg 100 g^−1^ DW.

### 2.4. Phenolic Profiling

The phenolic profile was determined following Concha-Meyer et al. [23] methodology with modifications. 350 mg of wine samples were mixed with 5 mL of methanol (75% *v*/*v*) (Merck, Darmstadt, Germany) and 100 µL of naringenin (internal standard) (Sigma-Aldrich, St. Louis, MO, USA). Then, the homogenized samples were centrifuged for 10 min at 11,180× *g* at 10 °C (International Equipment Company IEC Centra MP4R, Boston, MA, USA). After this, the supernatant was extracted and diluted with 20 mL of HPLC-grade water. Bond Elut C18 column (#12102052) (Agilent-Technologies, Little Falls, CA, USA) was prior hydrated with 1 mL methanol (75% *v*/*v*) and washed with 1 mL of HPCL water, to then be used to extract phenolics of samples. Samples were eluted through the column using 2 mL of pure 2n-propane (HPLC-grade Merck, Darmstad, Germany) and then, samples were dried using N_2_ gas in a gas blower concentrator (NB-503GBP, N-Biotec Co., Ltd., Seoul, Republic of Korea). Later, the samples were reconstituted by adding 200 µL of methanol (100% *v*/*v*) and then, the samples were sonicated using an ultrasonic bath (Sonorex Super TK52H, Bandelin, Berlin, Germany) for 1 min. Subsequently, the samples were transferred into a 150 µL vial insert that was immediately placed in an amber vial. Phenolic profiling was performed by using an UHPLC-MS (Dionex Ultimate 3000 + Exactive Plus™ Orbitrap, Thermo Scientific, Waltham, MA, USA) equipped with an electro-spray interface operating in negative ionization mode. Separation was achieved using an Hypersil Gold C18 column (Thermo Fisher Scientific, Bremen, Germany) 1.9 µm particle size (50 mm × 2.1 mm). Direct injection of 1 µL of the sample was performed using an autosampler at 4 °C. The solvents used were: (A) acetonitrile/water/formic acid (75.0:24.5:0.5% *v*/*v*), (B) acetonitrile/water/formic acid (5.0:94.5:0.5% *v*/*v*), establishing the following gradient 10% B from 0 to 1 min, 10% B from 1 to 5 min, 30% B from 5 to 10 min, 100% B from 10 to 18 min, 0% B from 18 to 24, at a flow of 300 µL min^−1^. Mass quantification conditions included negative ionization mode at 2500 spray voltage, vaporization temperature of 350 °C, sheath gas pressure of 40 arbitrary units (a.u.), auxiliary gas pressure 10 a.u. and capillary temperature of 35 °C. Collision gas used was N_2_ [24]. 

Data were processed using Xcalibur 2.1 software (Thermo Scientific, Bremen, Germany) for known standard assays using proper standards including hydrocinnamic acid, kaempferol, phloretin, quercetin, and resveratrol (*trans*-resveratrol) (1 mg mL^−1^ methanol) (Extrasynthese, Lyon, France) [23]. The quantification of the known compounds was carried out by means of a calibration curve. Calibration curve equation, R2, main ion mass (*m*/*z* ratio), and retention time (min) can be found in Concha-Meyer et al. [23]. All the values obtained were normalized to 350 mg DW, without maltodextrin, according to the percentage of loss of water and ethanol produced in the dealcoholizing process.

### 2.5. Experimental Design and Statistical Analysis

The variables were analyzed considering a completely randomized design with a factorial arrangement. The experimental design consisted of two factors considering wine varieties (Syrah and Listan Prieto) with four treatments (non-dealcoholized, dealcoholized, dehydrated by spray drying, and dehydrated by freeze drying). Each treatment consisted of two replicates by variety and type of wine, thereby a total of 16 replicates were performed in this trial. The variables were subjected to an analysis of variance (ANOVA) and significant differences were determined by Tukey’s test (*p*-value = 0.05) using Statgraphics Centurion XVII software (Statgraphics Technologies, Inc., The Plains, OH, USA).

## 3. Results and Discussion

### 3.1. Physicochemical, Color and Total Phenolic Content Analysis

Table 1 shows water activity (aw), pH and soluble solids (°Brix), and total phenolic content of non-dealcoholized, dealcoholized, spray-dried, and freeze-dried Listan Prieto and Syrah wine samples. The a_w_ values of dried treatments were significantly lower when compared to non-dealcoholized and dealcoholized samples, ranging from 0.14 to 0.24, therefore, preventing the development of pathogenic microorganisms which grow with a_w_ > 0.85 [25]. Even though ethanol concentration would be expected to lower the a_w_ [26], in this study, a_w_ of non-dealcoholized and dealcoholized showed no statistically significant differences (*p*-value ≥ 0.05). Low water activity in powders allows a free-flowing behavior since at values over 0.43 the powders are expected to show caking [21].

The soluble solids content of non-dealcoholized Syrah wines (7.10 °Brix) was significantly higher than Listan Prieto (4.20 °Brix), however, powdered wines showed no significant differences when wine varieties were compared. Listan Prieto is considered as a long-cycle variety and requires an elevated temperature to reach adequate technological maturity, whereas Syrah usually shows greater concentration of soluble solids when compared to most grape wine varieties [13,27]. In addition, drying treatments significantly increased soluble solids of Listan P Prieto and Syrah when compared to non-dealcoholized and dealcoholized wine samples. Maltodextrin encapsulation increases the solubility of hydrophobic compounds in water and other hydrophilic molecules such as amino acids, organic acids, and nucleic acid-related compounds [28]. 

pH values of freeze-dried samples showed to be significantly lower when compared to non-dealcoholized and spray-dried samples for both wine varieties (Table 1). According to the literature, processes such as freezing, and freeze-drying should not contribute to modifying pH within dried raw samples [29]. However, alcohol and water removal in wine by freeze drying result in a fraction constituted by glycerol, sugars, polyphenols, proteins, inorganic and organic salts, and organic acids, among others, that could contribute to a final lower pH [20]. In addition, cold generates the production of potassium bitartrate crystals in the stabilization process which induces a decrease in pH when they precipitate at a pH below 3.65 [30]. Low pH is responsible for tart and crisp taste and shelf-life extension in finished wine products and is also correlated with the color intensity of anthocyanins [31]. Anthocyanins capabilities to be used as food colorants are overshadowed by color variations due to pH-dependent reversible structural transformations, and processing and storage thermal instability [32]. Anthocyanins and other phenolic compounds encapsulation with carriers such as maltodextrin have been proven to be a good alternative to maintain and protect color over time [33].

Table 1 also shows the total phenolic content (TPC) of treated samples, where Syrah wines presented significantly higher TPC in spray-dried and freeze-dried samples when compared to Listan Prieto samples. Heras-Roger et al. [34] reported that Listan Prieto wines presented extremely lower anthocyanin, flavanol, and flavonol contents than several grapevine varieties, including Syrah. Furthermore, spray-dried samples showed higher TPC for both wine varieties when compared to freeze-dried samples. Davila-Trujillo et al. [35] performed nanoencapsulation of phenolic compounds from wine residues using spray drying, freeze-drying, and low-energy emulsion, observing that Syrah residue extract encapsulated by spray drying with maltodextrin had the highest TPC (4530.86 mg GAE 100 g^−1^ extract) among all the studied formulations. Also, other authors have observed that spray-dried products can present a higher retention of phenolic and flavonoid compounds when compared to freeze products in papaya pulp [36] and chokeberry [37]. High-temperature encapsulation may deactivate enzymes responsible for oxidative and hydrolytic reactions, therefore causing the prevention of polyphenols to decrease. Additionally, high-temperature thermal processing may produce cellular structure breakdown which causes unbinding of phenolic compounds, thus increasing presence and detection [38]. Spray drying is considered an encapsulation technology that is more suitable for industrial scale-up considering that it can be 4–5 times cheaper than freeze drying process ss [39].

Table 2 shows the color analysis results of dried Listan Prieto and Syrah wine samples. All color parameters showed significant differences (*p*-value < 0.05) for all the samples including the L* parameter, which indicates the luminosity of the product. Spray-dried Listan Prieto (66.25 ± 0.05) showed the highest L* value while freeze-dried Syrah had the lowest value (29.59 ± 0.08). Listan Prieto dried samples presented significantly higher L* levels than the Syrah ones. Heras-Roger et al. [40] reported that Listan Prieto wines presented the highest lightness (L* value) compared to the wines produced from several grapevine varieties, including Syrah. CIE Lab values a and b of the samples obtained by spray-dry were higher, which is reflected in a more intense chroma compared to the freeze-dry samples. The difference in the parameters a and b of the powdered wines can be due to the co-pigmentation phenomenon, which is generated when the anthocyanins present in wine bind non-covalently with phenolic compounds present in the two dealcoholized wine samples. Further, the increase in color intensity and color stability, resulting from co-pigmentation is due to a change in the balance of hydration towards flavylium forms (deep red) which are then trapped by the cofactors, towards quinone bases in the anthocyanin-co-pigment complexes (blue-purple colorations) [41]. This is important in the pH range of the pH of the wine where the hydrated forms of the anthocyanins, since the inclusion of flavylium cations in the complexes of co-pigmentation shifts the balance toward the formation of more cations flavylium [42].

**Figure 1:**
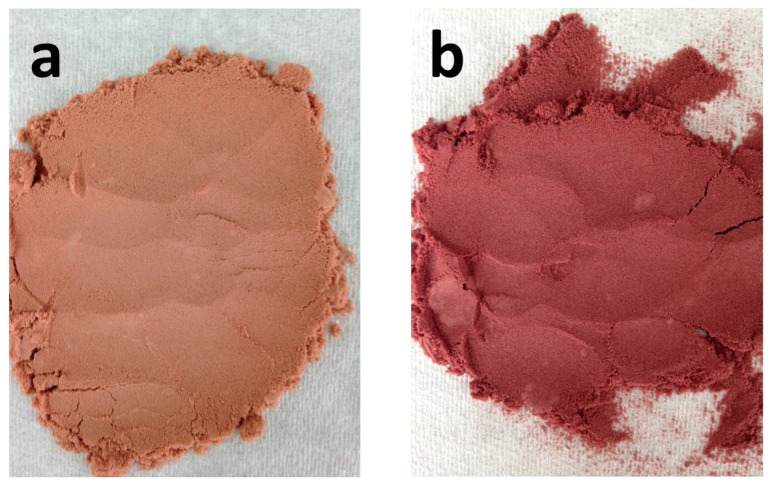
Maltodextrin encapsulated spray-dried Listan Prieto (**a**) and Syrah (**b**) wine samples.

Figure 1 shows spray-dried wine sample color differences and free-flowing powder appearance. Maltodextrin addition retained color since this carrier has white color and contributes to lightness in powder colors, which is linked to higher L* values [43].

### 3.2. Phenolic Profiling

Table 3 shows the concentration of *trans*-resveratrol, hydrocinnamic acid, phloretin, kaempferol, and quercetin found in Syrah and Listan Prieto treated wines. Wine variety did not affect the content of the studied metabolites in wine samples (*p*-value > 0.05). To our knowledge, there are scarce reports that evaluated phloretin and hydrocinnamic acid content in wines. Stilbenes, mainly resveratrol and viniferins are phytoalexins that are synthesized in the leaves and skin of grapes in response to abiotic stress [44]. Phenolic compounds are antioxidant compounds that contribute to the sensorial properties of wines [45,46]. Phenolic compound content in grapes varies mainly according to the variety, but nevertheless, in healthy grapes, *trans*-resveratrol content is scarce [47]. In this study, both cultivars and treatments showed similar contents of these phenolic compounds, probably because the samples were produced from healthy red grape varieties, which are vinified following a similar protocol.

Non-dealcoholized and dealcoholized samples showed significantly lower *trans*-resveratrol content when compared to spray-dried samples. Furthermore, dried samples presented significantly higher hydrocinnamic, kaempferol and quercetin concentrations when compared to non-dealcoholized and dealcoholized samples. Phloretin content was higher in non-dealcoholized and spray-dried samples than in dealcoholized samples. Spray-dried samples showed an increase of 152 and 357% in the *trans*-resveratrol content compared to initial wine samples. Freeze and spray-dried treatments when compared to initial wine samples produced an average increase of 209, 57, and 178% in hydrocinnamic acid, kaempferol, and quercetin concentration, respectively. Elimination of water and ethanol during spray and freeze drying allows the concentration of total phenolic compounds in wine powders [18] in this study to increase an average of 2.4 times when compared to non-dealcoholized samples. Moreover, some authors reported that the increase in water activity induces important losses of phenolic compounds on freeze-dried encapsulated wines, identifying water activity as a key factor affecting phenolic stability during storage [16,48].

In a hydroalcoholic solution, such as wine, the stability of resveratrol depends on light, temperature, and pH [49]. In this way, it has been possible to verify that *trans*-resveratrol is stable for months when it is protected from light, except at a pH greater than or equal to 10 [50]. Ultraviolet radiation shifts the isomeric equilibrium towards the formation of the *cis*-form of resveratrol [49,50]. The *cis*-isomer is only stable at pH close to neutrality, at low pH, it favors the cis form to isomerize to the trans, which is the most sterically stable form [50], this effect could explain the changes in the concentration of trans-resveratrol in dealcoholized wine, and powdered wine. Thus, the higher concentration of *trans*-resveratrol in the microencapsulated wine may be since in the freeze-drying process the wine was processed for a longer time and also exposed to ambient light, thus degrading the trans-resveratrol, a molecule that is highly oxidizable and which, according to Shi et al. [51], it has a half-life of between 30 and 45 min. This effect occurs to a lesser extent in the dry spray because the concentrated wine comes into contact with the maltodextrin in a shorter time, thus being protected from the aforementioned effect.

**Figure 2:**
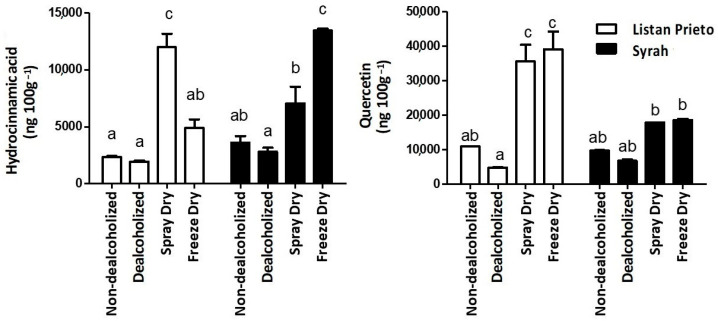
Interaction between wine variety and treatment (A × B) on hydrocinnamic acid and quercetin content in non-dealcoholized (control), dealcoholized and powdered wines made by freeze dry and spray dry from Listan Prieto (white bars) and Syrah (black bars) wine varieties. Different letters represent statistically significant differences among treatments and wine variety.

Figure 2 shows the interaction between variety and treatment factors on hydrocinnamic acid and quercetin content in Listan Prieto and Syrah wine samples. Listan Prieto wines powdered by spray dry and Syrah wines powdered by freeze dry showed significantly higher hydrocinnamic acid content when compared to the rest of the wine samples. Furthermore, Listan Prieto dried samples presented higher quercetin content than non-dealcoholized and dealcoholized samples, while quercetin of Syrah dried wines did not statistically differ from the other same type of wine samples. As previously mentioned, the freeze and spray drying process allowed the removal of water and ethanol from wine, and the use of maltodextrin as a drying aid led to an amorphous, glassy microstructure, in which the wine phenolic compounds of the dry wine extract were encapsulated [16]. Galmarini et al. [48] reported that encapsulation provided protection against conditions such as oxidation and thermal degradation. Based on the aforementioned, water activity and encapsulation provided stability on phenolic compounds compared to dealcoholized wine.

Samples obtained from the spray drying process were atomized and dried at high temperatures. Listan Prieto sprays dry samples presented higher hydrocinnamic acid and quercetin content than most of the rest of the studied treatments, except for Listan Prieto freeze dry (Figure 2). Ma et al. [52] reported an increase in phenolics due to the drying process at high temperatures that might be attributed to a release of bound phenolics from the tissue caused by drying. In this way, quercetin and ellagic acid were reported to present the highest thermal stability above most of the phenolic compounds [53]. Based on this, it is probable that the drying process in quercetin did not affect its content despite the freeze and high temperatures used in each of the drying processes. On the other hand, Listan Prieto dried samples presented higher content of quercetin than the Syrah dried samples (Figure 2). Despite that Listan Prieto grapes are characterized by their low phenolic content, Heras-Roger et al. [34] showed that flavonol proportion of myricetin and quercetin derivatives are higher in Listan Prieto than in Syrah. In addition, some individual flavonols were higher in Listan Prieto wines than in most of the other studied varieties, including Syrah [34,40]. In general, yields of all wine samples after encapsulation process varied from 78 to 85% with no statistically significant difference among variables.

## 4. Conclusions

Freeze-drying and spray-drying processes allowed the reduction of water activity in maltodextrin-encapsulated Listan Prieto and Syrah wines. Powdered wines were obtained presenting similar water activity and soluble solids. Furthermore, dried samples with both technologies showed no differences in *trans*-resveratrol, hydrocinnamic acid, phloretin, kaempferol, and quercetin content. In addition, powdered wines significantly concentrated hydrocinnamic acid and quercetin when compared to non-dealcoholized and dealcoholized wine samples. The present results show important data to consider drying technologies to concentrate and preserve antioxidant compounds of wine that can be used to formulate functional foods or nutraceutical products. Moreover, these results could be of potential importance for producers of Listan Prieto because they present an alternative for product diversification and add value to their wines.

The future perspectives of the powdered wine obtained in this work consist of evaluating the cardioprotective effect and carrying out studies on the shelf life and physical stability of the product obtained during its storage, to be able to use the powdered wine as a natural colorant in foods and/or to enriched foods with antioxidants such as anthocyanins and flavonoids.

## Figures and Tables

**Table 1:** Physicochemical parameters and total phenolic content of treated wine samples.

Wine Variety	Treatment	Water Activity (a_w_)	pH	Soluble Solids (°Brix)	Total Phenolic Content (mg GAE g^−1^)
Listan Prieto	Non-dealcoholized	0.93 ± 0.01 a	3.94 ± 0.07 a	4.20 ± 0.04 a	1.45 ± 0.07 a
	Dealcoholized	0.92 ± 0.01 a	3.48 ± 0.11 abc	5.20 ± 0.61 a	4.05 ± 0.07 a
	Spray Dry	0.17 ± 0.01 b	3.57 ± 0.16 ab	9.30 ± 0.18 c	41.70 ± 0.70 c
	Freeze Dry	0.24 ± 0.05 b	2.91 ± 0.27 c	9.00 ± 0.21 c	33.50 ± 0.57 b
Syrah	Non-dealcoholized	0.95 ± 0.01 a	3.66 ± 0.16 a	7.10 ± 0.12 b	1.85 ± 0.07 a
	Dealcoholized	0.92 ± 0.00 a	3.55 ± 0.14 ab	5.10 ± 0.19 a	5.45 ± 0.07 a
	Spray Dry	0.14 ± 0.03 b	3.62 ± 0.13 a	9.23 ± 0.11 c	51.40 ± 1.98 d
	Freeze Dry	0.24 ± 0.07 b	2.98 ± 0.14 bc	8.70 ± 0.21 c	40.35 ± 2.90 c

Mean plus standard deviation followed by different letters within each column do differ statistically (Tukey’s test, *p* ≤ 0.05).

**Table 2:** CIE Lab color parameters and HEX color code of dried wine samples.

Wine Variety	Treatment	L*	a	b	HEX Code	Color
Listan Prieto	Spray Dry	66.25 ± 0.05 d	45.04 ± 0.04 d	37.63 ± 0.04 d	#F97D60	
	Freeze Dry	59.41 ± 0.07 c	25.32 ± 0.06 c	24.72 ± 0.05 c	#C57D65	
Syrah	Spray Dry	37.10 ± 0.04 b	21.72 ± 0.08 b	13.89 ± 0.03 b	#7E4942	
	Freeze Dry	29.59 ± 0.08 a	8.58 ± 0.02 a	1.92 ± 0.05 a	#544143	

Mean plus standard deviation followed by different letter within each column do differ statistically (Tukey’s test, *p* ≤ 0.05).

**Table 3:** Content of metabolites (ng 100 g^−1^ DW) of treated wine samples.

	*Trans*-Resveratrol	Hydrocinnamic Acid	Phloretin	Kaempferol	Quercetin
**Wine variety (A)**					
Syrah	87.8 ± 30.7 a	6715.5 ± 2419.0 a	297.5 ± 37.4	2654.6 ± 456.4 a	13,238.3 ± 2946.0 a
Listan Prieto	82.5 ± 25.5 a	5273.5 ± 2324.2 a	336.2 ± 21.2	2718.6 ± 548.9 a	22,613.9 ± 8615.0 a
*p*-value	0.80	0.62	0.09	0.58	0.60
**Treatment (B)**					
Non-dealcoholized	60.6 ± 12.1 a	2961.2 ± 389.0 a	333.7 ± 23.0 b	2126.9 ± 151.0 b	10,344.5 ± 273.1 a
Dealcoholized	33.4 ± 5.3 a	2366.9 ± 252.3 a	244.1 ± 23.0 a	1585.6 ± 22.5 a	5806.1 ± 4499.2 a
Freeze Dry	93.7 ± 25.4 ab	9153.1 ± 1946.1 b	331.4 ± 30.0 ab	3700.8 ± 151.3 c	28,804.2 ± 5134.1 b
Spray Dry	152.8 ± 30.1 b	9496.9 ± 1388.1 b	358.3 ± 23.0 b	3333.23 ± 115.0 c	26,749.7 ± 467.0 b
*p*-value	0.00	0.00	0.013	0.00	0.00
**Interaction (A × B)**					
*p*-value	0.35	0.00	0.09	0.13	0.00

Significance (*p*-value) of wine variety (A), treatment (B), and A–B interactions. For a given factor and significance *p* < 0.05, different letters within a column represent significant differences (Tukey’s test, *p* < 0.05).

## Data Availability

Data is contained within the article.
